# Supplementary material for: Effect of Adding Bifidobacterium animalis BZ25 on the Flavor, Functional Components and Biogenic Amines of Natto by Bacillus subtilis GUTU09
Source: Foods. 2022 Sep 2;11(17):2674. doi: 10.3390/foods11172674 (PMC9455604; doi:10.3390/foods11172674)
Supplement: Supplementary file 1 [file foods-11-02674-s001.zip › foods-1846706-supplementary.pdf]

Table S1 Concentration of volatile substances in steamed soybean samples (SS), in soybean fermented by BZ25 (SFB), natto fermented by GUTU09 (NMB), and natto fermented by two-strain (NMBB)

Category	Name	Concentration(ug/L)			
		SS	SFB	NMB	NMBB
Amines	N,N-dimethyl-methylamine	-	-	161.65±14.32[a]	-
	Formamide	-	-	6.23±3.6[a]	-
	3-Methyl-butanamide	-	-	8.6±0.72[a]	-
	Ethosuximide	-	-	9.19±0.83[a]	-
Esters	Hexanoic acid ethyl ester	14.2±7[c]	29.77±4[c]	580.19±32.12[b]	5410.47±123.89[a]
	Octanoic acid ethyl ester	13.1±5[d]	366.07±21[a]	50.07±1.97[c]	255.57±24.42[b]
	Benzoic acid ethyl ester	115.5±32[a]	143.38±16[a]	-	9.42±1.37[b]
	2-Methyl-propanoic acid, ethyl ester	2858.2±100[a]	-	165.76±4.63[b]	172.33±21.38[b]
	3-Methyl-butanoic acid ethyl ester	-	-	-	12.99±0.71[a]
	Hexadecanoic acid methyl ester	-	14.22±3[b]	-	129.43±21.14[a]
	2-Methyl-butanoic acid ethyl ester	-	27±0.3[c]	83.77±5.47[b]	181.91±31.21[a]
	Heptanoic acid ethyl ester	-	29.47±2.5[b]	1.38±0.51[c]	77.64±23.15[a]
	Ethylphenyl acetate	6731.2±450[a]	1083.81±48[c]	4924.8±39.72[b]	539.57±31.25[d]
	2,2,4-Trimethyl-1,3-pentanediol diisobutyrate	-	-	17.78±3.42[a]	17.72±2.34[a]
	Benzeneacetic acid methyl ester	-	215.54±31[a]	187.38±32.26[a]	-
	Diethyl-acetic acid	-	-	2662.1±47.2[a]	-
	γ-Butyrolactone	441.2±45[a]	-	-	-
	Ethanedioic acid, diethyl ester	848.2±61[a]	-	-	-
	2-Butenoic acid, 2-propenyl ester	167.1±35[a]	-	-	-
	4-Isobutylbutanolide	-	400.82±34[b]	961.93±33.75[a]	6.27±0.35[c]

	Oxirane-2-carboxylic acid, ethyl ester	-	-	-	1100.91±45.23[a]
Pyrazines	Trimethyl-pyrazine	3833.8±233[c]	3452.16±231[c]	5938.15±135.23[b]	16423.69±345.23[a]
	2,5-Dimethyl-pyrazine	9363±600[b]	2377.69±123[c]	8822.82±219.78[b]	23981.63±421.8[a]
	2-Ethyl-3,5-dimethyl-pyrazine	16.9±3.2[c]	556.81±43[b]	1363.53±43.56[a]	72.35±22.32[c]
	2-Ethyl-5-methyl-pyrazine	-	128.38±21[b]	154.73±2.54[a]	119.1±13.43[b]
	Tetramethyl-pyrazine	-	825.9±31[c]	1610.35±41.24[b]	3474.49±123.23[a]
	Pyrazine	2054.1±89.2[a]	244.88±26[b]	-	19±2.34[c]
	Methyl-pyrazine	638±41[a]	-	278.95±31.24[b]	147.91±22.14[c]
	2-Ethenyl-6-methyl- pyrazine	-	2.17±0.5[b]	-	319±35.33[a]
	2,3,5-Trimethyl-6-ethylpyrazine	-	525.95±16[b]	156.93±13.26[c]	672.54±35.28[a]
Ketones	3-Hydroxy-2-butanone	292.4±50[d]	47154.9±567[a]	30944.69±750.37[b]	15988.37±332.89[c]
	5-Methyl-2-hexanone	73.5±21[c]	185.17±17[c]	1511.21±100.23[a]	970.81±67.25[b]
	2-Hexadecanone	88.2±18[c]	31.77±1.1[d]	241.02±21.32[b]	526.66±34.89[a]
	2,3-Butanedione	-	165.14±19[b]	-	3219.12±216.78[a]
	2-Nonanone	130.3±19[b]	1355.71±25.1[a]	1474.72±47.12[a]	1482.05±134.37[a]
	1-Phenyl-ethanone	50.8±12[c]	95.54±12.1[b]	36.07±2.12[c]	435.83±32.87[a]
	2-Heptanone	1297.1±46[b]	2376.09±89[a]	981.69±45.37[c]	-
	3-Octanone	783±49[a]	31.21±1.9[c]	223.71±33.42[b]	-
	2,3-Pentanedione	187.3±21[b]	334.78±17[a]	198.63±32.21[b]	-
	Acetone	27587.6±90[a]	17081.25±368.76[b]	7743.08±79.91[c]	-
	2-Hydroxy-3-pentanone	-	160.03±15.65[c]	675.69±32.27[a]	331.93±45.78[b]
	2,6-Dimethyl-4-heptanone	-	2.63±0.8[b]	177.17±27.17[a]	181.98±34.89[a]
	1-(4-Methylphenyl)-ethanone	46.8±10[a]	4.82±0.56[c]	-	15.63±2.35[b]
	3-Hydroxy-4-phenyl-2-butanone	-	-	6.14±3.24[b]	130.74±34.56[a]

	4-Methyl-3-penten-2-one	-	311.23±23.91[a]	109.12±12.11[b]	-
	2-Butanone	2219.7±145[b]	-	3875.48±72.67[a]	-
	3-Pentanone	8061±212[a]	-	-	-
	1-Octen-3-one	1089.7±134[a]	-	-	-
	6-Methyl-5-hepten-2-one	1065.1±146[a]	-	-	-
	3-Hydroxy-2-methyl-4H-pyran-4-one	-	-	-	2880.75±156.78[a]
	3-Hydroxy-3-methyl-2-Butanone	176.4±21[b]	41.6±3.42[bc]	-	1586.05±145.78[a]
	5-Methyl-2-heptanone	-	-	445.92±21.79[b]	961.25±35.67[a]
Acids	Acetic acid	6305.4±309[b]	9303.7±104.32[a]	3072.77±125.21[d]	3919.93±312.67[c]
	2-Ethyl-butanoic acid	-	-	2662.1±56.73[a]	682.54±43.47[b]
	2-Methyl-butanoic acid	886.8±52[c]	703.65±32.35[c]	3626.2±95.23[b]	5349.15±348.67[a]
	2-Methyl- Propanoic acid	242.7±31[c]	513.49±35.67[c]	2409.88±72.12[a]	2039.66±134.78[b]
	(R)-(-)-4-Methylhexanoic acid	-	17.42±2.13[bc]	39.05±2.89[b]	463.12±34.89[a]
	Octanoic acid	8.6±1[c]	89.8±3.2[b]	14.78±1.78[c]	112.23±18.93[a]
	Benzoic acid	7811±342[a]	6636.8±157.8[b]	2831.72±79.36[c]	191.82±18.98[d]
	Benzeneacetic acid	-	6.79±1.12[b]	12.89±1.79[b]	186.66±36.27[a]
	Octadecanoic acid	-	-	-	1037.58±127.89[a]
	n-Hexadecanoic acid	404.8±35[a]	-	310.8±21.9[b]	-
	3-Methyl-2-butenoic acid	-	33.16±2.47[b]	214.23±19.09[a]	-
	Hexanoic acid	344.9±21[b]	740.04±15.86[a]	114.02±16.43[c]	-
	Propanoic acid	-	980.37±35.42[a]	255.21±23.43[b]	-
	Formic acid	555±46[a]	64.21±12.21[b]	-	-
	3-Methyl-butanoic acid	885.5±67[b]	2265.9±36.72[a]	-	-
	4-Methyl-pentanoic acid	30.8±11[b]	-	111.68±17.34[a]	-

	4-Hydroxy-butanoic acid	-	296.84±16.21[a]	-	134.73±16.78[b]
Aldehydes	Decanal	551.2±25[b]	373.92±34.42[c]	-	667.46±35.45[a]
	Nonanal	482.3±22[b]	384.56±39.42[c]	125.14±14.32[d]	862.21±56.89[a]
	3-Methyl-butanal	15944±323[a]	-	501.05±45.35[b]	-
	2-Methyl-butanal	-	-	540.18±16.32[a]	-
	Octanal	284.7±21[a]	-	107.83±11.23[b]	-
	Phenylacetaldehyde	435.1±45[a]	163.6±14.75[b]	118.3±16.78[b]	-
	Furfural	1822.7±357[a]	264.79±24.86[b]	-	-
	Benzaldehyde	6799.8±345[a]	2084.12±46.77[b]	2212.72±45.37[b]	-
	Hexanal	4996.8±231[a]	142.14±13.96[b]	52.64±10.23[b]	-
	Heptanal	1297.1±127[a]	-	-	-
	2,4-Dimethyl-benzaldehyde	7.4±1.5[c]	296.84±21.76[c]	2.58±0.9[b]	388.14±45.53[a]
Alcohols	1-Octen-3-ol	1680.1±114[b]	128.04±13.25[c]	59.45±4.56[c]	9305.75±321.22[a]
	Trans-geraniol	-	228.8±21.24[a]	-	207.48±34.12[a]
	3-Octanol	-	128.04±13.42[b]	-	702.78±34.36[a]
	1-Hexanol	-	1763.54±128.37[a]	-	418.62±29.56[b]
	2-Ethyl-1-hexanol	-	171.26±4.39[b]	-	719.76±30.89[a]
	Ethanol	-	-	-	2490.17±320.37[a]
	1-Nonanol	-	-	118.3±21.23[a]	113.08±12.89[a]
	3-Ethyl-3-pentanol	-	-	-	44.14±12.29[a]
	2-Methyl-3-hexanol	-	-	854.58±23.67[a]	-
	1-Butanol	-	35.74±2.72[a]	10.84±0.85[b]	10.05±2.8[b]
	5-Methyl-2-hexanol	-	-	157.39±13.36[a]	44.08±11.9[b]
	Phenylethyl alcohol	-	-	111.35±18.34[b]	671.37±23.47[a]
	1-Hexadecanol	-	41.37±3.62[a]	-	49.41±23.43[a]

	2-Heptanol	-	111.84±12.43^{a}	-	12.22±2.33^{c}
	2,3-Butanediol	-	592.05±34.27^{b}	30.76±2.47^{c}	2787.28±34.26^{a}
	1-Octanol	-	-	29.66±3.66^{b}	303.27±17.31^{a}
	1-Nonen-3-ol	2212.7±45^{a}	-	-	-
	Benzyl alcohol	-	-	-	648.34±21.56^{a}
	2-Phenoxy-ethanol	-	-	356.24±23.67^{a}	52.32±13.42^{b}
	Eucalyptol	336.2±23^{a}	-	-	-
	(Z)-3-Methyl-2-pentenol	0^{b}	163.6±31.26^{a}	0^{b}	173.13±23.45^{a}
Aromatics	2-Methoxy-phenol	697.3±37^{c}	3300±109.27^{a}	854.18±51.47^{b}	742.97±34.56^{bc}
	Biphenyl	4.44±1.76^{b}	5.48±0.34^{b}	2.42±0.8^{b}	32.57±3.45^{a}
	Phenol	516.2±45^{c}	392.52±32.12^{d}	633.21±39.42^{b}	782.67±23.7^{a}
	p-Xylene	94.2±17^{b}	60.79±9.32^{c}	27.35±3.21^{d}	1255.45±23.45^{a}
	2,4-Di-tert-butylphenol	-	-	-	847.42±19.87^{a}
	4-Ethyl-phenol	28.1±11^{b}	-	186.68±21.34^{a}	-
	Toluene	1236.7±89^{a}	-	-	-
	Ethyl-benzene	682.6±61^{a}	-	8.37±3.37^{c}	591.68±35.34^{b}
	1,2-Dimethyl-benzene	-	165.14±14.62^{b}	-	1288.78±89.97^{a}
	1-Methyl-4-(1-methylethenyl)benzene	-	-	2366.46±134.39^{a}	348.25±23.89^{b}
Furans	2-Pentyl-furan	2910.2±35^{a}	728.92±19.22^{b}	204.75±12.22^{d}	451.85±34.65^{c}
	2,3-Dihydro-benzofuran	95.6±11^{c}	164.13±16.32^{b}	120.37±11.34^{c}	633.97±31.34^{a}
	2-ethyl-Furan	17697.6±198^{a}	4667.1±42.21^{b}	768.89±23.98^{c}	-
	5-Methyl-2-propylfuran	-	363.79±23.56^{b}	-	488.31±28.98^{a}
	2-Ethyl-5-methyl-furan	712±56^{a}	64.4±5.43^{b}	-	-
Others	Hexadecane	-	-	2078.36±67.35^{a}	1392.42±78.78^{b}

Undecane	-	-	917.59±35.63[a]	17.54±2.45[b]
Styrene	-	110.05±8.92[b]	-	1048.34±114.56[a]
(E)-1-Phenylbutene	-	-	-	15.09±3.45[a]
1-Heptadecene	-	-	-	29.54±3.78[a]
Naphthalene	2.07±0.8[b]	-	-	259.87±23.35[a]
Anethole	1354.8±35[b]	-	446.3±35.12[c]	2034.06±33.45[a]
1-Methyl-naphthalene,	-	271.24±23.24[a]	-	272.92±34.19[a]
Benzothiazole	24.9±4[b]	-	-	288.72±24.89[a]
1H-Indole	13.4±2[b]	3.76±0.32[c]	-	50.54±4.29[a]
Propanoic acid anhydride	-	-	-	2308.38±34.28[a]
Phenanthrene	-	174.55±14.52[a]	-	12.13±2.14[b]
Dimethyl Trisulfide	-	262.84±21.17[a]	26.53±2.35[b]	-
Maltol	1124.9±34[a]	-	-	-
Camphene	220±14[a]	72.3±0[b]	-	-
D-Limonene	639.5±39[a]	-	-	-
2-Methyl-thiophene	802.8±47[a]	-	-	-

Note: Means with Different different lower case letters (a–d) in the same row indicates a significant difference ($p < 0.05$).

Table S2 OAV of volatile substances identified in steamed soybean (SS), soybean fermented by BZ25 (SFB), natto made by GUTU09 (NMB), and natto fermented by two-strain (NMBB)

Number	Name	Aroma description	RI	ID	Threshold value (ug/L)[a]	OAV			
						SS	SFB	NMB	NMBB
	**Amines**								
A1	N,N-dimethyl-Methylamine	Fish	2081.3	MS, RI, Std	8-23[d]	-	-	7-20	-
A2	Formamide	Ammonium odour	1155.7	MS, RI	n.f.	-	-	-	-
A3	3-Methyl-butanamide	—	1917.7	MS, RI	n.f.	-	-	-	-
A4	Ethosuximide	—	2370.4	MS, RI	n.f.	-	-	-	-
	**Esters**								
B1	Hexanoic acid ethyl ester	Fruit, pineapple, banana aroma	1233.3	MS, RI	55.3[b]	< 1	< 1	10	98
B2	Octanoic acid ethyl ester	Cream, milk Fragrance	1436	MS, RI	13[c]	1	28	4	20
B3	Benzoic acid ethyl ester	Camomile, flower, celery, fruit	1676.6	MS, RI	0.6[d]	193	239	-	16
B4	2-Methyl-propanoic acid ethyl ester	Fruity	969	MS, RI	58[e]	49	-	3	3
B5	3-Methyl-butanoic acid ethyl ester	Fruity, apple-like,, strawberry-like	1068.2	MS, RI	7[c]	-	-	-	2
B6	Hexadecanoic acid methyl ester	Fruity, fermented pear-like	1334.2	MS, RI	4000000[f]	-	< 1	-	< 1
B7	2-Methyl-butanoic acid ethyl ester	Fruit, green, apples	1053.1	MS, RI	968-11700[g]	-	< 1	< 1	< 1
B8	Heptanoic acid ethyl ester	Green, diversileaf	1334.2	MS, RI	13200[h]	-	< 1	< 1	< 1

		artocarpus fruit							
B9	Ethylphenyl acetate	Flowery, fruit, cocoa fragrant	1793.5	MS, RI	4300[g]	< 1	< 1	1	< 1
B10	2,2,4-Trimethyl-1,3-pentanediol diisobutyrate	—	1879.9	MS, RI	4000[d]	-	-	-	< 1
B11	Benzeneacetic acid methyl ester	Jasmine flower, sweet, fruit	1767.6	MS, RI	0.16[i]	-	1347	1171	-
B12	Diethyl acetate	Sweet, pineapple, grape	1381.6	MS, RI	1500[f]	-	-	2	-
B13	γ-Butyrolactone	Milk, cream flavor	1636.5	MS, RI	35000[d]	< 1	-	-	-
B14	Ethanedioic acid diethyl ester	—	1537.9	MS, RI	n.f.	-	-	-	-
B15	2-Butenoic acid 2-propenyl ester	—	1128	MS, RI	n.f.	-	-	-	-
B16	4-Isobutylbutanolide	—	1377.9	MS, RI	n.f.	-	-	-	-
B17	Oxirane-2-carboxylic acid ethyl ester	—	1812.7	MS, RI	n.f.	-	-	-	-
	**Pyrazines**								
C1	Trimethyl-pyrazine	Nutty, cocoa-like, roasted	1407.7	MS, RI	50-96[g]	40-77	36-90	62-119	171-328
C2	2,5-Dimethyl-pyrazine	Cocoa, roasted nut, roast beef	1325.7	MS, RI	1700[j]	1	1	5	14
C3	2-Ethyl-3,5-dimethyl-pyrazine	Roasted potato	1465	MS, RI	7.5[b]	2	74	182	10
C4	2-Ethyl-5-methyl-pyrazine	Nutty	1394.4	MS, RI	16[k]	-	8	10	7
C5	Tetramethyl-pyrazine	Burnt coffee, grass	1478.3	MS, RI	730[c]	-	1	2	5
C6	Pyrazine	Rancid	1217.2	MS, RI	900[d]	2	< 1	-	< 1
C7	Methyl-pyrazine	Nutty, coffee, cocoa-	1270.6	MS, RI, Std	1900[d]	< 1	0	< 1	< 1

		like							
C8	2-Ethenyl-6-methyl-pyrazine	Caramel, nutty, roasted potato	1495.6	MS, RI	40[d]	-	< 1	-	8
C9	2,3,5-Trimethyl-6-ethylpyrazine	—	1516.3	MS, RI	n.f.	-	-	-	-
	**Ketones**								
D1	3-Hydroxy-2-butanone	Fat milk	1288.7	MS, RI	8000[g]	< 1	6	4	2
D2	5-Methyl-2-hexanone	—	1141.8	MS, RI	62-89[d]	1	2-3	17-24	11-16
D3	2,3-Hexadecanone	Butter caramel fruit	2079.1	MS, RI	7.3[d]	12	4	33	72
D4	2,3-Butanedione	Sweet cream	984.7	MS, RI	5[e]	-	33	-	644
D5	2-Nonanone	Sweet coconut	1342.7	MS, RI	32[d]	4	42	46	46
D6	1-Phenyl-ethanone	Almond	1664	MS, RI	65[k]	< 1	1	< 1	7
D7	2-Heptanone	Mild medicinal fragrance	1181.3	MS, RI	1[j]	1287	2376	982	-
D8	3-Octanone	Herb, butter	1251.9	MS, RI	1.3[g]	602	24	172	-
D9	2,3-Pentanedione	Caramel, nuts	1061.6	MS, RI	30[d]	6	11	7	-
D10	Acetone	irritating flavors	819.8	MS, RI	1100[d]	25	16	7	-
D11	2-Hydroxy-3-pentanone	Truffle, earth, nuts	1361.7	MS, RI	3000[d]	-	< 1	< 1	< 1
D12	2,6-Dimethyl-4-heptanone	—	1391	MS, RI	9300[d]	-	< 1	< 1	< 1
D13	1-(4-Methylphenyl)-ethanone	—	1789.7	MS, RI	21[d]	2	< 1	-	< 1
D14	3-Hydroxy-4-phenyl-2-butanone	—	2275.8	MS, RI	750-1000[d]	-	-	< 1	< 1
D15	4-Methyl-3-penten-2-one	—	1132.8	MS, RI	200[k]	-	2	< 1	-
D16	2-Butanone	Fruit green	905.3	MS, RI	1300[g]	2	-	3	-

D17	3-Pentanone	Almond, malt, pungent	918.9	MS, RI	3000[d]	3	-	-	-
D18	1-Octen-3-one	—	1301.2	MS, RI	1000[d]	1	-	-	-
D19	6-Methyl-5-hepten-2-one	Pepper, mushroom	1338.9	MS, RI	68[d]	16	-	-	-
D20	3-Hydroxy-2-methyl-4H-pyran-4-one	Caramel-like	1977	MS, RI, Std	2500[d]	-	-	-	1
D21	3-Hydroxy-3-methyl-2-butanone	—	1244.6	MS, RI, Std	n.f.	-	-	-	-
D22	5-Methyl-2-heptanone	—	1256.4	MS, RI	n.f.	-	-	-	-
	**Acids**								
E1	Acetic acid	Stimulative sour	1451.1	MS, RI	13-150[g]	13-485	-	62-715	26-301
E2	2-Ethyl-butanoic acid	Fruit milchigs	1380.5	MS, RI	93-460[d]	-	-	6-29	1-7
E3	2-Methyl-butanoic acid	Fruit, sour, cheese	1667.7	MS, RI	2200[d]	< 1	< 1	2	2
E4	2-Methyl-propanoic acid	Pungent, rancid	1565.5	MS, RI	1580[c]	< 1	< 1	2	1
E5	(R)-(-)-4-Methylhexanoic acid	—	1923.5	MS, RI	7300[d]	-	< 1	< 1	< 1
E6	Octanoic acid	Rancidify	2056.3	MS, RI	2700[c]	< 1	< 1	< 1	< 1
E7	Benzoic acid	Perfume pungent odor	2444.6	MS, RI	1000[g]	8	7	3	< 1
E8	Benzeneacetic acid	Flowery, animal fragrance	2564.1	MS, RI	1430[b]	-	< 1	< 1	< 1
E9	Octadecanoic acid	—	2719.4	MS, RI	20000[d]	-	-	-	< 1
E10	n-Hexadecanoic acid	—	2199.2	MS, RI	>100000d	< 1	-	-	-
E11	3-Methyl-2-butenoic acid	—	1796.9	MS, RI	14000[d]	-	< 1	< 1	-
E12	Hexanoic acid	Sour, sweaty spicy	1845.6	MS, RI	2520[g]	< 1	< 1	< 1	-
E13	Propanoic acid	Stimulative sour smell	1538.5	MS, RI	1000[d]	-	< 1	< 1	-

E14	Formic acid	Penetrating odor	1502.5	MS, RI	46000[d]	< 1	< 1	-	-
E15	3-Methyl-butanoic acid	Stimulative rancid odor	1670.2	MS, RI	490[d]	2	5	-	-
E16	4-Methyl-pentanoic acid	Rancid	1803.4	MS, RI	144[c]	< 1	-	< 1	-
E17	4-Hydroxy-butanoic acid	—	1645.6	MS, RI, Std	n.f.	-	-	-	-
	**Aldehydes**								
F1	Decanal	Sweet, flowery wax fragrance	1502	MS, RI	0.1[j]	5510	3739	-	6675
F2	Nonanal	flowery citrus, fat wax fragrant	1396.1	MS, RI	3.1[g]	151	124	40	278
F3	3-Methyl-butanal	Malt,unpleasant smell	919.7	MS, RI	31.6[d]	505	-	16	-
F4	2-Methyl-butanal	Cocoa, almond	916.3	MS, RI	1[d]	-	-	540	-
F5	Octanal	Crude oil smell	1284.6	MS, RI	0.9[k]	316	-	120	-
F6	Phenylacetaldehyde	Fengxinzi taste	1647	MS, RI	4[k]	108	41	30	-
F7	Furfural	Sweet, bread, caramel-like aroma	1466.3	MS, RI	44000[c]	< 1	< 1	-	-
F8	Benzaldehyde	Cherry nuts, bitter almond aroma	1528.8	MS,RI, Std	85[g]	80	25	26	-
F9	Hexanal	Grass, fat	1082.9	MS, RI	230[g]	22	< 1	< 1	-
F10	Heptanal	Strong fruit aroma	1182.9	MS, RI	260[d]	5	-	-	-
F11	2,4-Dimethyl-benzaldehyde	—	1830.5	MS, RI	n.f.	-	-	-	-
	**Alcohols**								

G1	1-Octen-3-ol	Mushroom green fragrance	1447.3	MS, RI	10[g]	168	13	6	931
G2	Trans-geraniol	Flowery, lemon aroma	1845.3	MS, RI	1[f]	-	208	-	189
G3	3-Octanol	Herbaceous, melon, citrus-like odor	1389.8	MS, RI	18[d]	-	7	-	39
G4	1-Hexanol	Green, fowery	1350.3	MS, RI	34[g]	-	52	-	12
G5	2-Ethyl-1-hexanol	Rose, green	1486.5	MS, RI	200[g]	-	< 1	-	4
G6	Ethanol	Sweet, wine flavor	937.2	MS, RI	620[g]	-	-	-	4
G7	1-Nonanol	Fat green aroma	1656.7	MS, RI	45.5[d]	-	-	3	2
G8	3-Ethyl- 3-pentanol	—	1370.3	MS, RI	42-84[d]	-	-	-	0-1
G9	2-Methyl-3-hexanol	—	1370.8	MS, RI	46-81[d]	-	-	11-19	-
G10	1-Butanol	Spicy wine	1146.9	MS, RI	2730[c]	-	< 1	< 1	< 1
G11	5-Methyl-2-hexanol	—	1276.1	MS, RI	330-650[d]	-	-	< 1	< 1
G12	Phenylethyl alcohol	Honey, rose	1918.4	MS, RI	12-21[g]	-	-	< 1	< 1
G13	1-Hexadecanol	—	2374	MS, RI	1100[d]	-	< 1	-	< 1
G14	2-Heptanol	Mushroom	1316.3	MS, RI	1430[h]	-	< 1	-	< 1
G15	2,3-Butanediol	Fruit, onion	1538.5	MS, RI	>100000[g]	-	< 1	< 1	< 1
G16	1-Octanol	Rose, citrus aroma	1554.4	MS, RI	1100[h]	-	-	< 1	< 1
G17	1-Nonen-3-ol	—	1532.4	MS, RI	1300[d]	-	-	-	-
G18	Benzyl alcohol	Sweet, flower	1882.6	MS, RI	40900[c]	-	-	-	< 1
G19	2-Phenoxy-ethanol	—	2151.9	MS, RI	690000[f]	-	-	< 1	< 1

G20	Eucalyptol	—	1206.7	MS, RI, Std	4.6[d]	73	-	-	-
G21	(Z)-3-Methyl-2-pentenol		1320.3	MS, RI, Std	n.f.	-	-	-	-
	**Aromatics**								
H1	2-Methoxy-phenol	Spicy medicine fragrance	1869.1	MS, RI	1.5[g]	465	2200	569	495
H2	Biphenyl	—	2005	MS, RI	0.5[d]	9	11	5	65
H3	Phenol	Phenol	2010.6	MS, RI	21[g]	25	19	30	37
H4	p-Xylene	Plastic, pungent	1137.5	MS, RI	1000[i]	< 1	< 1	< 1	1
H5	2,4-Ditert-butylphenol	—	2306.8	MS, RI	500[d]	-	-	-	2
H6	4-Ethyl-phenol	Musty	1213.1	MS, RI	51[d]	< 1	-	4	-
H7	Toluene	Paint	1040.8	MS, RI	527[d]	2	-	-	-
H8	Ethyl-benzene	—	1121.9	MS, RI	2205[j]	< 1	-	< 1	< 1
H9	1,2-Dimethyl-benzene	Geranium	1182.8	MS, RI	n.f.	-	-	-	-
H10	1-methyl-4-(1-methylethenyl)benzene	—	1455.4	MS, RI	n.f.	-	-	-	-
	**Furans**								
I1	2-Pentyl-furan	Bean, green	1230.1	MS, RI	5.8[j]	502	126	35	78
I2	2,3-Dihydro-benzofuran	—	2399	MS, RI	48[d]	2	3	3	13
I3	2-Ethyl-furan	Malt fragrance	957.3	MS, RI	2.3[j]	7694	2029	334	-
I4	5-Methyl-2-propylfuran	—	1577.9	MS, RI	n.f.	-	-	-	-
I5	2-ethyl-5-methyl-Furan	—	1035.1	MS, RI	n.f.	-	-	-	-
	**Others**								
J1	Hexadecane	Alkane	1405	MS, RI, Std	25[j]	-	-	84	56
J2	Undecane	Alkane	1090.5	MS, RI, Std	1578[j]	-	-	< 1	< 1

J3	Styrene	Balsamic, gasoline	1259.6	MS, RI	65[d]	2	2	-	16
J4	(E)-1-Phenylbutene	—	1519.6	MS, RI	150[d]	-	-	-	< 1
J5	1-Heptadecene	—	2578.4	MS, RI	8000[d]	-	-	-	< 1
J6	Naphthalene	—	1757.7	MS, RI	6[d]	-	-	-	43
J7	Anethole	Fennel, spicy Licorice smell	1839	MS, RI	100[f]	-	-	4	20
J8	1-Methyl-naphthalene	Naphthalene camphor aroma	1870.8	MS, RI	58.1[j]	5	5	-	5
J9	Benzothiazole	Coffee, meat flavor	1975	MS, RI	80[d]	-	-	-	4
J10	1H-Indole	Mothball, burnt, flowery	2461.7	MS, RI	40[i]	< 1	< 1	-	1
J11	Propanoic acid anhydride	—	1800.5	MS, RI	3400[d]	-	-	-	< 1
J12	Phenanthrene	—	2750.4	MS, RI, Std	55-60[d]	3	3	-	< 1
J13	Dimethyl trisulfide	Sulfuric, strong onion odor	1380.1	MS, RI	14[g]	19	19	2	-
J14	Maltol	Sweet malt aroma	2069.6	MS, RI	210[d]	5	-	-	-
J15	Camphene	Camphor	1066	MS, RI	26000-30000[d]	< 1	< 1	-	-
J16	D-Limonene	Lemon, citrus, sweet	1189.7	MS, RI	n.f.	-	-	-	-
J17	2-Methyl-thiophene	Sulfur	1091.6	MS, RI	n.f.	-	-	-	-

Note: a: Odor thresholds were determined in water. Odor thresholds were taken from reference. [b] Odor thresholds taken from Ref. [1]; [c] Odor thresholds taken from Ref. [2]; [d] Odor thresholds taken from Ref. [3]; [e] Odor thresholds taken from Ref. [4]; [f] Odor thresholds taken from Ref. [5]; [g] Odor thresholds taken from Ref. [6]; [h] Odor thresholds taken from Ref. [7]; [i] Odor thresholds taken from Ref. [8]; [j] Odor thresholds taken from Ref. [9]; [k] Odor thresholds taken from Ref. [10]. OAV: Odor activity value; ID: Identification basis; MS: mass spectrometry; RI: retention index; Std: authentic standards; "n.f.", "—":Data was not found in literatures;

References

1. Zheng, Y.; Sun, B.; Zhao, M.; Zheng, F.; Huang, M.; Sun, J.; Sun, X.; Li, H. Characterization of the key odorants in chinese Zhima aroma-type Baijiu by gas chromatography-olfactometry, quantitative measurements, aroma recombination, and omission studies. **2016**, *64*, 5367-5374. https://doi.org/10.1021/acs.jafc.6b01390.
2. Li, H.; Qin, D.; Wu, Z.; Sun, B.; Sun, X.; Huang, M.; Sun, J.; Zheng, F. Characterization of key aroma compounds in Chinese Guojing sesame-flavor Baijiu by means of molecular sensory science. **2019**, *284*, 100-107. https://doi.org/10.1016/j.foodchem.2019.01.102.
3. L.J. van Gemert, Z. Complilations of odour threshold values in air, water and other media[M]. The Netherlands: Oliemans Punter & Partners BV. **2003**.
4. Sun, J.; Li, Q.; Luo, S.; Zhang, J.; Huang, M.; Chen, F.; Zheng, F.; Sun, X.; Li, H. Characterization of key aroma compounds in Meilanchun sesame flavor style baijiu by application of aroma extract dilution analysis, quantitative measurements, aroma recombination, and omission/addition experiments. **2018**, *8*, 23757-23767. https://doi.org/10.1039/c8ra02727g.
5. Shi, J.; Tong, G.; Yang, Q.; Huang, M.; Ye, H.; Liu, Y.; Wu, J.; Zhang, J.; Sun, X.; Zhao, D. Characterization of key aroma compounds in tartary buckwheat (fagopyrum tataricum gaertn.) by means of sensory-directed flavor analysis. **2021**, *69*, 11361-11371. https://doi.org/10.1021/acs.jafc.1c03708.
6. Chen, Y.; Li, P.; Liao, L.; Qin, Y.; Jiang, L.; Liu, Y. Characteristic fingerprints and volatile flavor compound variations in Liuyang Douchi during fermentation via HS-GC-IMS and HS-SPME-GC-MS. **2021**, *361*, 130055. https://doi.org/10.1016/j.foodchem.2021.130055.
7. Zhao, D.; Shi, D.; Sun, J.; Li, A.; Sun, B.; Zhao, M.; Chen, F.; Sun, X.; Li, H.; Huang, M., et al. Characterization of key aroma compounds in Gujinggong Chinese Baijiu by gas chromatography-olfactometry, quantitative measurements, and sensory evaluation. **2018**, *105*, 616-627. https://doi.org/10.1016/j.foodres.2017.11.074.
8. Guo, X.; Ho, C.-T.; Schwab, W.; Wan, X. Effect of the roasting degree on flavor quality of large-leaf yellow tea. **2021**, *347*, 129016. https://doi.org/10.1016/j.foodchem.2021.129016.
9. Gu, S.-q.; Wang, X.-c.; Tao, N.-p.; Wu, N. Characterization of volatile compounds in different edible parts of steamed Chinese mitten crab (Eriocheir sinensis). **2013**, *54*, 81-92. https://doi.org/10.1016/j.foodres.2013.05.018.
10. Zhu, J.; Niu, Y.; Xiao, Z. Characterization of the key aroma compounds in Laoshan green teas by application of odour activity value (OAV), gas chromatography-mass spectrometry-olfactometry (GC-MS-O) and comprehensive two-dimensional gas chromatography mass spectrometry (GC x GC-qMS). **2021**, *339*, 128136. https://doi.org/10.1016/j.foodchem.2020.128136.
